# Subjective Taste and Smell Changes in Conjunction with Anxiety and Depression Are Associated with Symptoms in Patients with Functional Constipation and Irritable Bowel Syndrome

**DOI:** 10.1155/2021/5491188

**Published:** 2021-09-18

**Authors:** Jie Liu, Chaolan Lv, Dandan Wu, Ying Wang, Chenyu Sun, Ce Cheng, Yue Yu

**Affiliations:** ^1^Department of Gastroenterology, the First Affiliated Hospital of USTC, Division of Life Sciences and Medicine, University of Science and Technology of China, Hefei, Anhui 230001, China; ^2^South District of Endoscopic Center, the First Affiliated Hospital of USTC, Division of Life Sciences and Medicine, University of Science and Technology of China, Hefei, Anhui 230001, China; ^3^Internal Medicine, AMITA Health Saint Joseph Hospital Chicago, 2900 N. Lake Shore Drive, Chicago, 60657 Illinois, USA; ^4^The University of Arizona College of Medicine at South Campus, 2800 E Ajo Way, Tucson, 85713 AZ, USA

## Abstract

**Background:**

Patients with functional constipation (FC) and irritable bowel syndrome (IBS) often report psychological abnormalities and decreased eating enjoyment. Several patients also complain of changes in the sense of smell and taste, but these are often disregarded clinically.

**Aims:**

Therefore, there is a need to determine whether taste/smell disturbances and psychological abnormalities are present in patients with FC or IBS and whether these are related to the severity of lower gastrointestinal symptoms.

**Methods:**

A total of 337 subjects were recruited, including FC (*n* = 115), IBS (*n* = 126), and healthy controls (*n* = 96). All participants completed questionnaires evaluating taste and smell (taste and smell survey (TSS)), Lower Gastrointestinal Symptoms Rating Scale (LGSRS), Hamilton anxiety scale (HAMA), and Hamilton depression scale (HAMD). TSS recorded information on the nature of taste and smell changes (TSCs) and the impact of these changes on the quality of life. LGSRS was used to assess the severity of lower gastrointestinal symptoms; HAMA and HAMD scales were used to reflect the psychosocial state. This study protocol was registered on the Chinese Clinical Trial Registry (No. ChiCTR-2100044643).

**Results:**

Firstly, we found that taste and smell scores were higher in patients with IBS than in healthy controls. Secondly, for FC and IBS patients, LGSRS was significantly correlated with the taste score (Spearman′s rho = 0.832, *P* < 0.001). LGSRS was also significantly correlated with HAMA (Spearman′s rho = 0.357, *P* = 0.017) and HAMD (Spearman′s rho = 0.377, *P* = 0.012). In addition, the taste score was significantly correlated with HAMD (Spearman′s rho = 0.479, *P* = 0.001), while the smell score was also significantly correlated with HAMD (Spearman′s rho = 0.325, *P* = 0.031). Thirdly, 60.87% and 71.43% of patients complained of taste abnormality, while 65.22% and 71.43% had smell abnormality in the FC and IBS groups, respectively. Meanwhile, 47.83% and 47.62% of patients suffered from anxiety, while 43.48% and 57.14% suffered from depression in the FC and IBS groups, respectively. Finally, we found significant differences in the taste, smell, HAMD, and LGSRS scores between the female and male IBS groups (*P* < 0.050).

**Conclusions:**

TSCs and psychological disorders are prominent in FC and IBS patients. Taste abnormalities, as well as anxiety and depression, are significantly correlated with LGSRS. Awareness of this high prevalence of taste/smell abnormalities and the psychological changes among patients with FC and IBS may help better predict and understand the severity of symptoms.

## 1. Introduction

It is recognized that an increasing number of patients are suffering from chronic gastrointestinal diseases worldwide [1, 2]. In a recent survey, 40.0% of patients with gastroparesis and 60.0% of patients with gastroesophageal reflux disease (GERD) scored their taste abnormalities as either mild to moderate or severe to intolerable, respectively [3]. Kabadi et al. [3] found significant abnormalities in taste and smell in patients with gastroparesis and GERD, and this change was significantly correlated with upper gastrointestinal symptoms. In patients with inflammatory bowel disease, the smell function was significantly lower than the general population, even though patients themselves were not aware of their reduced olfactory/gustatory function [4]. Therefore, it is necessary to understand the relationship between chemical sensation and digestive tract symptoms.

Disorders in smell and taste were reported to occur more frequently in the elderly, and such disorders influence nutrition, safety, quality of life, and physical and mental health [5]. Bernhardson et al. [6] reported that TSCs caused pain and reduced quality of life. Because dining is not only for nutrition intake but also has important symbolic, cultural, and religious values that may affect the psychological and social aspects of life. TSCs of patients may result in chronic unconscious weight loss and malnutrition, secondary to the change of food enjoyment, dietary preferences, and dietary intake [7, 8]. Therefore, the severity of lower gastrointestinal symptoms and characteristics of TSCs and psychological abnormality should be assessed to better understand and support patients.

It is worth noting that depression may result or cause gustatory and olfactory dysfunction. TSCs may cause negative emotions in cancer patients, including disappointment, depression, and sadness [9, 10]. One previous study analyzed the data obtained from a nationally representative probability sample of 3005 older American adults and reported an association between any depressive symptoms and olfactory dysfunction [11]. Moreover, a meta-analysis study evaluated the relationship between depression and TSCs, and the authors concluded that depressed individuals had more deterioration of olfaction than nondepressed controls [12]. Kohli et al. [13] included 10 studies and demonstrated that patients with depression had reduced olfactory performance compared with the healthy controls. Conversely, patients with olfactory dysfunction have symptoms of depression that worsen with the severity of smell loss. Another U.S. population-based survey detected a strong association between major depression and alterations in smell and taste [14]. On the other hand, globus sensation, a functional gastrointestinal disease, often arises concomitantly with psychological comorbidities, including major depression [15]. However, most of the previous studies on TSCs and chronic digestive diseases had ignored the effect of psychological comorbidities [3, 4].

In this study, taste and smell survey (TSS) was used to evaluate TSCs in patients with functional constipation (FC) and IBS, while Lower Gastrointestinal Symptoms Rating Scale (LGSRS) was used to assess the severity of lower digestive tract symptoms. The Hamilton anxiety scale (HAMA)/Hamilton depression scale (HAMD) was then used to measure the psychological state of the patients. This study compared the TSCs, the severity of lower gastrointestinal symptoms, and the scores of patients' anxiety and depression scale with those of healthy controls. In addition, we further explored the association among TSCs, the severity of lower gastrointestinal symptoms, and the anxiety and depression scale's scores.

## 2. Methods

### 2.1. Patients

Patients (outpatients and inpatients) who met the Rome IV diagnostic criteria [16] for FC and IBS were diagnosed by a gastroenterologist (Y Y) experienced in the diagnosis of functional digestive disorders and were recruited into the study at the Center of Gastrointestinal Motility, the First Affiliated Hospital of University of Science and Technology of China from January 2018 to August 2020. The inclusion criteria of FC and IBS were used based on the Rome IV criteria [17–19]. Meanwhile, colonoscopy or barium enema was performed within the first four weeks, and organic intestinal lesions have been excluded.

The exclusion criteria included the following: (i) severe heart and lung diseases, diabetes, nephropathy, and other chronic diseases associated with neuropathy or gastrointestinal disorders such as ulcers, cancer, and esophageal varices; (ii) patients who were taking drugs that could affect mental status within one month such as antidepressants, corticosteroids, or sedative-hypnotics; and (iii) participants who had diseases that could affect taste and smell sensation, including patients with malignant tumors who were taking antitumor drugs, as well as allergic patients who were taking antiallergic drugs and so on.

This study protocol was registered on the Chinese Clinical Trial Registry (No. ChiCTR-2100044643), and the study was approved by the Institutional Review Board of the First Affiliated Hospital of University of Science and Technology of China (Ethics Committee of the First Affiliated Hospital of University of Science and Technology of China). In addition, all patients signed a written informed consent before being included in the study.

115 patients with FC and 126 patients with IBS were collected as research objects surveyed in the Anhui Provincial Hospital from January 2018 to August 2020. Meanwhile, 96 healthy subjects were recruited from accompanying persons of included patients after advertisement in the First Affiliated Hospital of University of Science and Technology of China and were considered the control group.

### 2.2. Demographics Questionnaire

The patients were subjected to a questionnaire which asked about their age, gender, and length of diagnosis with FC and IBS. Participants' weight, in kilograms, divided by their height, in meters squared, was used for calculating body mass index (BMI). Subjects also listed all current medicines, tests that had been done, past medical conditions, and past surgeries.

### 2.3. Taste and Smell Survey

Taste and smell survey was compiled by Heald et al. [16] for HIV patients. There were 9 questions related to taste change and 5 questions related to smell change. TSS was widely used to assess changes in taste and smell in cancer patients [20–22]. The taste survey consisted of 9 items. Subjects were asked whether there was a change in taste self-perception (stronger or weaker) in salty, sweet, sour, and bitter tastes and the extent of this change (insignificant, mild to moderate, and severe to intolerable). 8 items scored 0-1 points and 1 item scored 0-2 points, with a total score of 10 points. The smell survey consisted of 5 items. Subjects were asked whether there was a change in smell self-perception, whether a particular food smells stronger or weaker and the extent of this change (insignificant, mild to moderate, and severe to intolerable). 4 items scored 0-1 points and 1 item scored 0-2 points, with a total score of 6 points. For all the questions, subjects were asked to compare their taste and smell senses at the time of data collection to the time before they were diagnosed with FC and IBS. Healthy controls were asked to compare their taste and smell senses to 10 years prior. The internal consistency reliability of previous search report was 0.89 [22].

### 2.4. Lower Gastrointestinal Symptoms Score

LGSRS was revised according to the gastrointestinal symptom rating scale [23]. There were 15 questions in the original score table, including five aspects of gastrointestinal symptoms: abdominal pain (including abdominal pain, nausea, and vomiting), reflux symptoms (including heartburn and acid reflux), diarrhea, symptoms of dyspepsia (including diarrhea, loose stool, fecal incontinence, and sense of urgency in defecation), symptoms of indigestion (including bellyache, abdominal distention, belching, and increased exhaust), and symptoms of constipation (including constipation, hard stool, and incomplete defecation). Each question provided four choices, and each symptom is scored separately from light to heavy, with a score of 0-3: 0 (asymptomatic), 1 (mild symptoms), 2 (moderate symptoms), and 3 (severe symptoms), and finally, each item's score was summed to obtain a total score. The higher the score, the more serious the gastrointestinal symptoms were. According to the above contents, 10 indicators, including periumbilical pain, diarrhea, constipation, loose stool, fecal incontinence, incomplete defecation, lower abdominal distention, abnormal bowel sounding, increased exhaust, and hard stool, were selected as the indicators of the lower gastrointestinal symptoms rating scale (LGSRS), with a cumulative score of LGSRS. All the scales were completed independently within 10 to 15 minutes and were guided by doctors trained in a unified way to review the symptoms within one week. Cronbach's alpha coefficients were used for measuring the internal consistency reliability and validity of LGSRS. In this study, Cronbach's alpha = 0.72, indicating that our LGSRS had fair internal consistency and reliability.

### 2.5. Investigation of Mental State

The Hamilton anxiety scale (HAMA) [24] was used to assess the mental state of the patients, which consisted of two subscales: psychic anxiety and somatic anxiety. The HAMA contains 14 items, and each item is rated on a 5-point Likert scale ranging from grade 0 to grade 4, which refers to no symptom and extremely severe symptoms, respectively. A global HAMA score of >6 indicates ‘anxiety.'

In addition, the Hamilton depression scale (HAMD) contains 17 items that were grouped into five structural factors: “anxiety/somatization,” “mental disorders,” “retardation symptoms,” “sleep disturbances,” and “weight loss.” HAMD uses a 5-point Likert scale ranging from grade 0 to grade 4, referring to no symptoms and extremely severe symptoms, respectively. A global HAMA score of >7 indicates ‘depression' [25].

Three specially trained professionals administered HAMA and HAMD through conversation and observation.

### 2.6. Statistical Analysis

The EpiData 3.1 software (The Epidata Association, Odense, Denmark) was used to input data, while the SPSS21.0 software (SPSS Inc., Chicago, IL) was used for statistical analysis. The normal distribution data is expressed as means ± SEM, while the data with nonnormal distribution were represented by the median and range. Continuous variables were compared parametrically using Student's *t*-test or nonparametrically using the Mann–Whitney *U* test. Moreover, prevalence was calculated using mean ± 2SD of healthy controls as the upper limit of normal, while Spearman's correlation was used to correlate the different scores. Cronbach's alpha was calculated to measure the internal consistency reliability and validity of LGSRS. The difference was statistically significant with *P* < 0.05.

## 3. Result

### 3.1. Demographics Data

Among the 115 FC patients, the average age was 37.87 ± 8.11 years old, which included 60 males and 55 females; among the 126 IBS patients, the average age was 36.52 ± 8.83 years old, which include 54 males and 72 females; among the 96 healthy controls, the average age was 35.63 ± 8.37 years old, which include 45 males and 51 females. According to Rome IV criteria [17, 19], there were 88 IBS-diarrhea patients, 35 IBS-constipation patients, and 3 IBS-mixed patients. No significant differences in age, sex, and BMI were detected between the three groups, see Table 1.

### 3.2. Different Scores in the 3 Groups

The taste score in the 3 groups (healthy controls, FC, and IBS) was 0.88 ± 0.81, 3.13 ± 1.46, and 3.57 ± 1.57, respectively. The smell score in the 3 groups (healthy controls, FC, and IBS) was 0.63 ± 0.50, 2.09 ± 1.24, and 2.00 ± 0.95, respectively. This revealed a significant difference in taste and smell scores between patients with FC/IBS and the control group. The HAMA score in the 3 groups (healthy controls, FC, and IBS) were 3.19 ± 1.76, 9.56 ± 5.10, and 9.19 ± 4.23, respectively. The HAMD score in the 3 groups (healthy controls, FC, and IBS) was 3.19 ± 1.80, 10.04 ± 4.38, and 9.71 ± 4.93, respectively. There was a significant difference in the HAMA score and HAMD score between patients with FC/IBS and the control group. The LGSRS score in the 3 groups (healthy controls, FC, and IBS) was 2.31 ± 1.40, 15.52 ± 3.10, and 14.52 ± 3.57, respectively. Thus, we found a significant difference in LGSRS scores between patients with FC/IBS and the control group (Table 2).

### 3.3. Correlation in Different Scores

The obtained results indicated that LGSRS was significantly correlated with the taste score (Spearman′s rho = 0.832, *P* < 0.001) in the overall patients' group. LGSRS was significantly correlated with HAMA (Spearman′s rho = 0.357, *P* = 0.017) and HAMD (Spearman′s rho = 0.377, *P* = 0.012). In addition, the taste score was significantly correlated with HAMD (Spearman′s rho = 0.479, *P* = 0.001), while the smell score was also significantly correlated with HAMD (Spearman′s rho = 0.325, *P* = 0.031). All the above results are represented in Table 3.

### 3.4. Taste Survey of the TSS

In the taste survey, 35 and 42 patients of the FC and IBS group found their tastes were different from before, respectively. Interestingly, patients often found that they had a weaker taste of saltiness, sweetness, and sourness; in contrast, the taste of bitterness was stronger. 10 patients with FC reported severe to intolerable change in their sense of taste, while 6 patients with IBS found severe to intolerable change in their gustatory senses (Table 4).

### 3.5. Smell Survey of the TSS

In the smell survey, 50 patients with FC found their foods smelled different from before, while 48 IBS patients found that their foods smelled different compared to the past. Interestingly, patients often felt their sense of smell diminished. 10 patients with FC found severe to intolerable changes in their sense of smell, while 18 patients with IBS found severe to intolerable alterations in their olfactory senses (Table 5).

### 3.6. Prevalence of Taste/Smell Abnormalities

Prevalence was calculated using mean ± 2SD of healthy controls as the upper limit of normal. Taste scores (out of 10 points) of healthy controls were 0.88 ± 0.81, and thus, the upper limit of normal was 2.50. Similarly, the upper limit of the smell score was 1.63. Therefore, there were 6 (6.25%), 70 (60.87%), and 90 (71.43%) participants who had taste abnormality, while 0 (0.00%), 75 (65.22%), and 90 (71.43%) participants had smell abnormality in the control group, FC group, and IBS group, respectively. Notably, there were more patients with taste abnormality in the FC (*χ*^2^ = 67.733, *P* <0.001) and IBS (*χ*^2^ = 94.311, *P* <0.001) groups compared to healthy controls. Similarly, there were more patients with smell abnormality in FC (*χ*^2^ = 97.136, *P* < 0.001) and IBS (*χ*^2^ = 115.325, *P* < 0.001) than healthy controls.

### 3.7. Prevalence of Anxiety and Depression

Generally, a global HAMA score of >6 indicates ‘anxiety,' while a global HAMD score of >7 indicates ‘depression.' There were 6 (6.25%), 55 (47.83%), and 60 (47.62%) patients who suffered from anxiety, while 0(0.00%), 50(43.48%), and 72 (57.14%) patients reached the depression criteria in the healthy control group, FC group, and IBS group, respectively. Notably, the number of patients with anxiety in the FC (*χ*^2^ = 44.007, *P* < 0.001) and IBS (*χ*^2^ = 44.635, *P* < 0.001) groups was more than in healthy controls. Similarly, there were more patients with depression in the FC (*χ*^2^ = 54.702, *P* < 0.001) and IBS (*χ*^2^ = 81.189, *P* < 0.001) groups than in healthy patients.

### 3.8. Comparative Analysis of Different Scores between Male and Female

The taste, smell, HAMD, and LGSRS scores in the IBS female group were 4.08 ± 1.26, 2.17 ± 1.15, 11.25 ± 5.36, and 15.17 ± 4.02, respectively; while the taste, smell, HAMD, and LGSRS scores in the IBS male group were 2.89 ± 1.61, 1.78 ± 0.42, 7.67 ± 3.01, and 12.44 ± 3.98, respectively. There were significant differences in the taste, smell, HAMD, and LGSRS scores between the female and male IBS groups (*P* < 0.050). However, the taste, smell, HAMA, HAMD, and LGSRS scores did not differ in female FC patients compared to male patients (Table 6).

## 4. Discussion

The chemical senses of taste and smell are essential to life. Physiological TSCs alert us to dangers (e.g., gas leaks), prevent us from ingesting toxins, and support our oral nutrition [26]. However, pathologically TSCs might contribute to an increased risk of malnutrition (under- or overnutrition) [27], low mood, diminished social interaction, and reduced quality of life [28]. This study shows that taste and smell abnormalities in patients with FC and IBS were not uncommon. Importantly, the taste abnormalities were positively correlated with the severity of lower gastrointestinal symptoms. A breakdown of individual taste complaints showed that most patients noted their gustatory functions were weaker when eating salty, sweet, and sour food items but were stronger when tasting bitter food, consistent with previous results reported by Silke et al. [4]. Additionally, patients often suffer from mental disorders of anxiety and depression, and higher HAMA score/HAMD scores were positively correlated with the severity of lower gastrointestinal symptoms. Notably, the HAMD score was positively correlated with the smell and taste scores of all subjects in the study. Interestingly, we compared the difference of evaluated characteristics in the current study and found that female IBS patients reported more severe alterations in taste and smell, lower gastrointestinal symptoms, and depression when compared with male patients, which is similar to a previous study [29]. However, taste and smell changes did not differ in female FC patients compared to male patients.

Presently, the mechanism of the changes in taste and smell caused by FC and IBS symptoms has not yet been fully elucidated, but these may be related to abnormal brain-gut axis interactions [30–32]. The brain-gut axis is recognized as a complex bidirectional communication system between the gastrointestinal tract and the brain, with many cellular and molecular pathways acting along this axis [33]. Kidd et al. [34] confirmed that enterochromaffin cells might be a luminal sensor for odorants and taste molecules followed by regulating gut motility. Steinbach et al. [35] demonstrated that scores of odor threshold were decreased while the scores of odor identification and odor discrimination were increased in IBS patients compared to the control, supporting the idea of a central etiology of IBS. Furthermore, a previous review concluded that altered gut microbiota in IBS produced a complex interaction with genetic variants dispersed in the human genome (intrinsic factors) and led to individual epigenetic prints, including many of the genes and molecular mechanisms involved in taste biology [36, 37]. Meanwhile, studies have also found that the morphology and metabolism of the insula in depressive patients were significantly altered. The insular lobe is a cortical structure located deep in the brain tissue and participates in processing taste and smell [38, 39]. Aschenbrenner et al. [40] found that anorexic patients had a significant decrease in taste and olfactory function, which was associated with higher depression scores. This study is the first to demonstrate that a large number of patients with FC and IBS are afflicted by TSCs as well as anxiety and depression, which is in line with previous studies [41, 42]. In addition, we detected a positive association between depression score and TSCs as well as the severity of lower gastrointestinal symptoms.

So far, there is a lack of definitive biomedical solutions to alleviate or restore taste and smell functions and adjust individuals' TSCs [42]. Studies have reported how patients find ways to avoid unpleasant taste or smell experiences or manage the social impact of TSCs [43]. Based on patients' understanding of the TSCs, proactive patients with a positive attitude will take the initiative to manage their eating habits, such as eating healthier (less fat, more vegetables), choosing seasonings to adjust the taste of food, avoid odor, to name a few [44]. Therefore, FC and IBS patients need to be aware of the potential changes in their taste and smell to be able to manage their eating habits. Meanwhile, physicians should also pay more attention to this phenomenon because our study shows that this may help better predict and understand the severity of lower gastrointestinal symptoms and TSC's association with psychiatric comorbidity and symptoms.

## 5. Limitations

There following are some shortcomings in this study: (1) The study used food-related smell and taste scores rather than objective tests [45, 46], giving rise to inherent recall bias that may result in participants underscoring or exaggerating their senses and feelings unconsciously. (2) Our sample size was quite small; therefore, larger-scale and multicenter studies are warranted in the future. Consequently, the difficulty of helping patients with taste and smell changes underscores the need of a diagnostic and evaluation tool for taste and smell changes and evidence-based treatment strategies. Meanwhile, the relationship among TSCSs, LGSRS, and psychological comorbidities is still unclear and needs further research clinically.

## 6. Conclusions

Taste and smell changes are common but are often the neglected symptoms in patients with digestive disorders. In this study, we found that changes in taste and smell were common in FC and IBS patients. The change in patients' taste is related to their LGSRS, and these patients were more likely to suffer from anxiety and depression, which were also found to be significantly correlated with LGSRS. Awareness of this high prevalence of taste/smell abnormalities and the consequent psychological alterations among patients with FC and IBS may also help better understand the severity of their lower gastrointestinal symptoms. Therefore, more high-quality evidence is warranted to guide future clinical practice.

## Figures and Tables

**Table 1:** Demographics and characteristics of the three groups (healthy controls, functional constipation, and irritable bowel syndrome) in this study.

	Overall (*N* = 337)	Healthy controls (*n* = 96)	FC (*n* = 115)	IBS (*n* = 126)	*χ*^2^/*t*	*P*
Gender						
Male (*n*)	159	45	60	54	2.099	0.350
Female (*n*)	178	51	55	72		
Age (years, mean ± SE)	45.71 ± 10.64	35.63 ± 8.37	37.87 ± 8.11	36.52 ± 8.83	1.744	0.087
BMI (kg/m^2^, mean ± SE)	21.31 ± 4.03	21.53 ± 3.67	20.45 ± 3.05	21.68 ± 4.83	0.789	0.434

FC: functional constipation; IBS: irritable bowel syndrome; body mass index (BMI) = weight (in kg)/height^2^ (in m^2^). No statistically significant difference was noted in age, gender, and BMI.

**Table 2:** Taste, smell, HAMA, and HAMD scores and lower gastrointestinal symptoms rating score in the 3 study groups (healthy controls, functional constipation, and irritable bowel syndrome).

	Healthy controls (*n* = 96)	FC (*n* = 115)	IBS (*n* = 126)
Taste score (out of 10 points) (mean ± SD)	0.88 ± 0.81	3.13 ± 1.46	3.57 ± 1.57
*P* value vs. healthy controls		*t* = 5.61, *P* < 0.001	*t* = 6.24, *P* < 0.001
Smell score(out of 6 points) (mean ± SD)	0.63 ± 0.50	2.09 ± 1.24	2.00 ± 0.95
*P* value vs. healthy controls		*t* = 5.26, *P* < 0.001	*t* = 4.46, *P* < 0.001
HAMA(out of 64 points) (mean ± SD)	3.19 ± 1.76	9.56 ± 5.10	9.19 ± 4.23
*P* value vs. healthy controls		*t* = 4.79, *P* < 0.001	*t* = 5.33, *P* < 0.001
HAMD (out of 72 points) (mean ± SD)	3.19 ± 1.80	10.04 ± 4.38	9.71 ± 4.93
*P* value vs. healthy controls		*t* = 5.90, *P* < 0.001	*t* = 5.03, *P* < 0.001
LGSRS (out of 30 points) (mean ± SD)	2.31 ± 1.40	15.52 ± 3.10	14.52 ± 3.57
*P* value vs. healthy controls		*t* = 15.89, *P* < 0.001	*t* = 12.90, *P* < 0.001

FC: functional constipation; IBS: irritable bowel syndrome; LGSRS: lower gastrointestinal symptoms rating scale.

**Table 3:** Correlation among taste, smell, HAMA, HAMD, and LGSRS scores for functional constipation and irritable bowel syndrome patients.

	FC (*n* = 115)		IBS (*n* = 126)		Overall patients (*n* = 241)	
	Spearman's rho	*P*	Spearman's rho	*P*	Spearman's rho	*P*
Taste vs. LGSRS	0.796	<0.001	0.890	<0.001	0.832	<0.001
Smell vs. LGSRS	0.046	0.834	0.702	<0.001	0.271	0.075
HAMA vs. LGSRS	0.010	0.964	0.627	0.002	0.357	0.017
HAMD vs. LGSRS	0.103	0.641	0.585	0.005	0.377	0.012
Taste vs. smell	0.088	0.690	0.672	0.001	0.319	0.035
Taste vs. HAMA	0.177	0.419	0.673	0.001	0.260	0.088
Taste vs. HAMD	0.232	0.288	0.690	0.001	0.479	0.001
Smell vs. HAMA	0.790	<0.001	0.605	0.004	0.185	0.230
Smell vs. HAMD	0.807	<0.001	0.339	0.132	0.325	0.031

FC: functional constipation; IBS: irritable bowel syndrome; LGSRS: lower gastrointestinal symptoms rating scale. Data are expressed as Spearman's correlation coefficient rho with *P* values.

**Table 4:** Breakdown of the detailed taste complaints from the taste and smell survey.

		Healthy controls (*n* = 96)	FC (*n* = 115)	IBS (*n* = 126)
Foods taste different than they used to				
Yes		12 (12.50)	35 (30.43)	42 (33.33)
No		84 (87.50)	80 (69.57)	84 (66.67)
I have noticed a change in my sense of taste				
Yes		6 (6.25)	30 (26.09)	36 (28.57)
No		90 (93.75)	85 (73.91)	90 (71.43)
I have a persistent bad taste in mouth				
Yes		6 (6.25)	45 (39.13)	60 (47.62)
No		90 (93.75)	70 (60.87)	66 (52.38)
Drugs interfere with my sense of taste				
Yes		0 (0.00)	40 (34.78)	48 (38.10)
No		96 (100.00)	75 (65.22)	78 (61.90)
Comparison of sense of taste now to before diagnosis				
I have noticed a change in salt				
Yes	Salt tastes stronger	6 (6.25)	10 (8.70)	18 (14.29)
	Salt tastes weaker	6 (6.25)	40 (34.78)	36 (28.57)
	Cannot taste	0 (0.00)	5 (4.35)	6 (4.76)
No		84 (87.50)	60 (52.17)	66 (52.38)
I have noticed a change in sweet				
Yes	Sweet tastes stronger	12 (12.50)	10 (8.70)	18 (14.29)
	Sweet tastes weaker	6 (6.25)	20 (17.39)	36 (28.57)
	Cannot taste	0 (0.00)	0 (0.00)	0 (0.00)
No		78 (81.25)	85 (73.91)	72 (57.14)
I have noticed a change in sour				
Yes	Sour tastes stronger	0 (0.00)	15 (13.04)	12 (9.53)
	Sour tastes weaker	12 (12.50)	20(17.39)	30 (23.81)
	Cannot taste	0 (0.00)	5 (4.35)	6 (4.76)
No		84 (87.50)	75 (65.22)	78 (61.90)
I have noticed a change in bitter				
Yes	Bitter tastes stronger	6 (6.25)	20 (17.39)	36 (28.57)
	Bitter tastes weaker	6 (6.25)	15 (13.04)	24 (19.05)
	Cannot taste	0 (0.00)	5 (4.35)	6 (4.76)
No		84 (87.50)	75 (65.22)	60 (47.62)
Rate abnormal sense of taste				
Insignificant		90 (93.75)	80 (69.57)	96 (76.19)
Mild to moderate		6 (6.25)	25 (21.74)	24 (19.05)
Severe to intolerable		0 (0.00)	10 (8.70)	6 (4.76)

Data are expressed as *n* (%). FC: functional constipation; IBS: irritable bowel syndrome.

**Table 5:** Breakdown of the detailed smell complaints from the taste and smell survey.

	Healthy controls (*n* = 96)	FC (*n* = 115)	IBS (*n* = 126)
I have noticed a change in my sense of smell			
Yes	12 (12.50)	50 (43.48)	48 (38.10)
No	84 (87.50)	65 (56.52)	78 (61.90)
Foods smell different than they used to			
Yes	12 (12.50)	55 (47.83)	54 (42.86)
No	84 (87.50)	60 (52.17)	72 (57.14)
Specific drugs interfere with my sense of smell			
Yes	6 (6.25)	30 (26.09)	36 (28.57)
No	90 (93.75)	85 (73.91)	90 (71.43)
I have noticed a change in odors			
Stronger	12 (12.50)	25 (21.74)	18 (14.28)
Weaker	6 (6.25)	40 (34.78)	24 (19.05)
No change	78 (81.25)	50 (43.48)	84 (66.67)
Rate abnormal sense of smell			
Insignificant	84 (87.50)	85 (73.91)	72 (57.14)
Mild to moderate	12 (12.50)	20 (17.39)	36 (28.57)
Severe to intolerable	0 (0.00)	10 (8.70)	18 (14.29)

Data are expressed as *n* (%). FC: functional constipation; IBS: irritable bowel syndrome.

**Table 6:** Gender-related differences of taste, smell, HAMA, HAMD, and LGSRS scores in FC and IBS patients.

	FC (*N* = 115)				IBS (*N* = 126)			
	Female (*n* = 55)	Male (*n* = 60)	*t*	*P*	Female (*n* = 72)	Male (*n* = 54)	*t*	*P*
Taste score	3.32 ± 1.49	3.07 ± 140	0.55	0.583	4.08 ± 1.26	2.89 ± 1.61	4.67	<0.001
Smell score	1.89 ± 0.86	2.21 ± 0.73	1.41	0.163	2.17 ± 1.15	1.78 ± 0.42	2.37	0.020
HAMA	9.90 ± 3.13	9.18 ± 3.28	1.19	0.210	9.42 ± 4.55	8.86 ± 2.82	1.07	0.168
HAMD	10.36 ± 3.11	9.56 ± 3.47	0.97	0.332	11.25 ± 5.36	7.67 ± 3.01	4.41	<0.001
LGSRS	14.21 ± 4.02	13.89 ± 3.14	0.54	0.589	15.17 ± 4.02	12.44 ± 3.98	3.77	<0.001

FC: functional constipation; IBS: irritable bowel syndrome; LGSRS: lower gastrointestinal symptoms rating scale.

## Data Availability

All data generated or analyzed during this study are included in this published article. The data used to support the findings of this study are available from the corresponding author upon request. Data were collected by authorized researchers.
